# Lattice doping regulated interfacial reactions in cathode for enhanced cycling stability

**DOI:** 10.1038/s41467-019-11299-2

**Published:** 2019-08-01

**Authors:** Lianfeng Zou, Jianyu Li, Zhenyu Liu, Guofeng Wang, Arumugam Manthiram, Chongmin Wang

**Affiliations:** 1 Environmental Molecular Sciences Laboratory, Pacific Northwest National Laboratory, 3335 Innovation Boulevard, Richland, WA 99354 USA; 2 McKetta Department of Chemical Engineering & Texas Materials Institute, The University of Texas at Austin, Austin, TX 78712 USA; 3 Department of Mechanical Engineering and Materials Science, University of Pittsburgh, Pittsburgh, PA 15261 USA; 4 Materials Science and Engineering Program and Texas Materials Institute, The University of Texas at Austin, Austin, TX 78712 USA

**Keywords:** Energy science and technology, Materials science, Materials for energy and catalysis, Batteries, Electrochemistry

## Abstract

Interfacial reactions between electrode and electrolyte are critical, either beneficial or detrimental, for the performance of rechargeable batteries. The general approaches of controlling interfacial reactions are either applying a coating layer on cathode or modifying the electrolyte chemistry. Here we demonstrate an approach of modification of interfacial reactions through dilute lattice doping for enhanced battery properties. Using atomic level imaging, spectroscopic analysis and density functional theory calculation, we reveal aluminum dopants in lithium nickel cobalt aluminum oxide are partially dissolved in the bulk lattice with a tendency of enrichment near the primary particle surface and partially exist as aluminum oxide nano-islands that are epitaxially dressed on the primary particle surface. The aluminum concentrated surface lowers transition metal redox energy level and consequently promotes the formation of a stable cathode-electrolyte interphase. The present observations demonstrate a general principle as how the trace dopants modify the solid-liquid interfacial reactions for enhanced performance.

## Abstract (web summary)

The electrode/electrolyte interface plays a critical role in a Li-ion battery. Here the authors report that Al doping can tailor the interfacial reactions to lead to enhanced structural stability and cyclability of cathode. Al dopants form not only lattice solid solution but also Al_2_O_3_ islands on the surface.

## Introduction

Applications of Li ion batteries in many advanced fields, such as long distance driving electrical vehicles and grid energy storage, require the cathode materials with high-energy density and stable cycling. Extensive efforts, either searching for new materials or modifying the existing ones, have been devoted to high-energy cathodes for commercial batteries^1–5^. The nickel–manganese–cobalt (NMC)-based layered materials fall into one of the categories that are capable of meeting the high demand from electromobility fields because of their high theoretical specific energy and low cost^6–8^. From the point of view of specific capacity, one of the most effective strategies is to push the upper limit of Ni content in the Ni-rich variants. Theoretically, with the increase of Ni concentration from 33% to >90%, the specific capacity increases from 160 to >220 mAhg^−1^^9,10^. However, high Ni concentration often leads to poor cycling stability, which is largely associated with the side reactions occurring at the electrode–electrolyte interface. On one hand, the electrochemical cycling results in the progressive formation of Ni^2+^ on the particle surfaces, which consequently leads to the several deterioration effects: (1) layered to spinel or rock salt phase transition that retards the Li ions diffusivity and gives rise to the cell impedance^11–13^; (2) accelerating the dissolution of transition metals (TMs), which deposits on the anode and causes capacity decay^14,15^; (3) contributing to the formation of intergranular cracks in the secondary particles and inherently affects the long-term cycling stability^16–18^. On the other hand, the TM redox couples at highly charged state may react with electrolyte, referred as to “parasitic reactions”, and causes the failure of cells^19,20^.

One of the most prevalent strategies for resolving the cycling instability, that is associated with cathode structural instability, is cationic doping. Intensive experimental work, often in trial and error, have been performed by selecting different dopants, such as Al, Mg, K, Na, Fe, W, and Zr, which all demonstrate improvement in terms of cycling stability^9,21–28^. In particular, Al is the one that has been widely deployed in the commercial Ni-rich cathodes. The addition of Al into the Ni and Co oxide-based layered structure endows the cathode with numerous beneficial effects, such as improved structural stability, thermal properties, and long-term cycling stability^17,29–31^. However, a high concentration of Al simultaneously reduces the reversible capacity because Al is an inactive component for electrochemical cycling. Therefore, a limited amount of Al within 5 at% is usually considered to offer the most optimized performance^32–34^. In general, the prevailing knowledge about doping effects are limited in the context of bulk properties, because the majority of the dopants is well-incorporated in the bulk and alloyed with the parent phase. However, the sole knowledge obtained from bulk remain far from sufficient to understand the Al function for accounting the observed dramatic improvements of structural and cycling stability.

Here we use two Ni-rich cathodes, one with the formula of LiNi_0.94_Co_0.06_O_2_ (NC) and the other with an addition of 2 at% Al in the NC materials (LiNi_0.92_Co_0.06_Al_0.02_O_2_, termed as NCA), to elucidate the enhanced interfacial and cycling stability of NCA by Al doping. We reveal that the Al dopant enhances cycling performance from two aspects: improve the electrode structural stability and modify the cathode electronic property and thus reactivity. Our scanning transmission electron microscopy (STEM) observations coupled with energy-dispersive x-ray spectroscopy (EDS) mappings reveal that Al is partitioned into two parts: as lattice solid solution with an Al concentration of <2 at% at the core of the primary particle and an enriched concentration of 6 at% on the primary particle surface; and as Al_2_O_3_ nano-islands that cover ~1% of primary particle surfaces. The atomistic simulations reveal that the Al-concentrated shell facilities the formation of phosphate-containing cathode electrolyte interface (CEI) layers as a consequence of the dopant induced down shift of Fermi level. The CEI layers, coupled with the sparsely scattered Al_2_O_3_ nano-islands, plays a dominant role for enhancing the battery performance.

## Results

### Properties of pristine materials

**Fig. 1:**
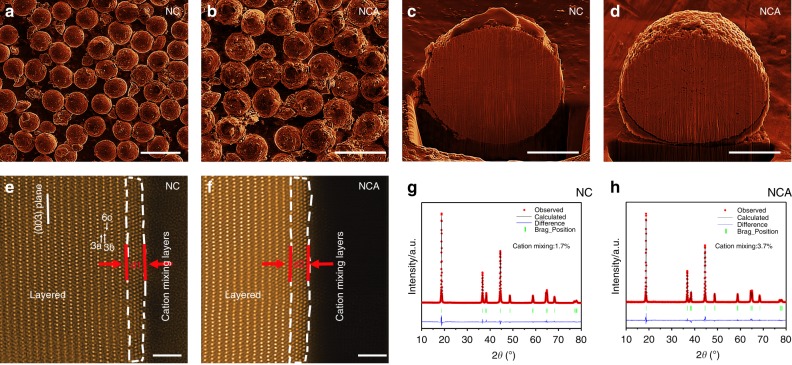
Surface and bulk characterizations of NC and NCA pristine materials. **a**, **b** SEM images of NC and NCA from top view, showing the secondary particles are of spherical shape with narrow size distribution. **c**, **d** SEM images of NC and NCA secondary particle from cross-sectional view. **e**, **f** Atomic resolution HAADF-STEM images of NC and NCA, seen from [100] zone axis. The white dashed lines outline the cation mixing regions on primary particle surfaces. d1 and d2 represent the thickness of cation mixing layers in NC and NCA, respectively. **g**, **h** XRD patterns of NC and NCA with Rietveld refinement analysis, the fitted results are presented in Supplementary Table 1. Scale bar, 20 μm **a**, **b**, 4 μm **c**, **d**, and 20 μm **e**, **f**

The structural and morphological homogeneity of the as-prepared layered materials are evaluated by using a combination of scanning electron microscopy (SEM), STEM, and x-ray diffraction (XRD). The SEM images in Fig. 1a and b display the typical macroscopic morphology and size distribution of pristine NC and NCA cathode, respectively. Both materials are composed of spherical secondary particles with a uniform size of ~12 μm in diameter. Given the pulverization processes are often accompanied with disintegration inside secondary particles and well-recognized to deteriorate the battery performance^12,18,35,36^, several randomly picked secondary particles are sliced into halves using focused ion beam (FIB) and the assembly features inside the buried bulk are examined. The cross-sectional SEM image in Fig. 1c captures the internal structure of NC particles, which is featured with a limited amount of small cavities and no prominent cracks are observed. In contrast, the cavity density in NCA (Fig. 1d) is slightly higher than that in NC. High angle annular dark field (HAADF)-STEM image, seen along the [010] zone axis, demonstrates that the primary particle surface of the as-synthesized NC materials possesses a cation mixing layer with a thickness of ~1 unit cell, and the structure from second unit cell maintains the well-defined layered symmetry, with the lithium, oxygen, and TM ions located in the 3a, 6c, and 3b sites, respectively, as pointed by the white arrows (Fig. 1e and Supplementary Fig. 2). Similar surface reconstruction layers with a unit cell thickness are observed for the case of pristine NCA as shown in Fig. 1f.

In addition to the local diagnosis, the powder XRD was employed to reveal the ensemble-averaged crystal structure of raw materials at bulk level. Figure 1g, h are the XRD patterns of both pristine materials, where all the evident signals can be conclusively registered to the characteristic peaks of layered structure, confirming the NC and NCA materials are well-prepared without introducing a secondary phase or impurities. The Rietveld refinement analysis of NC yields a lattice parameter of *a* = *b* = 2.871 Å, and *c* = 14.176 Å, which is in line with reported layered structure for the Ni-rich cathodes^9,17,37^. Compared to NC, the NCA shares the same R$\bar 3$m symmetry while undergoes a slight lattice expansion in the **c** direction and shrinkage in the **a** direction (Supplementary Table 1), resembling to the lattice evolution during Li extraction, yet with a reduced amount^38^. Moreover, the NC shows a minor degree of cation mixing of 1.7%, suggesting the amount of anti-site Ni ions, originated from the Ni^2+^ migration from TM layers to Li layers, is well controlled. For NCA, the cation mixing value is 3.7%.

### Electrochemical performance of NC and NCA

**Fig. 2:**
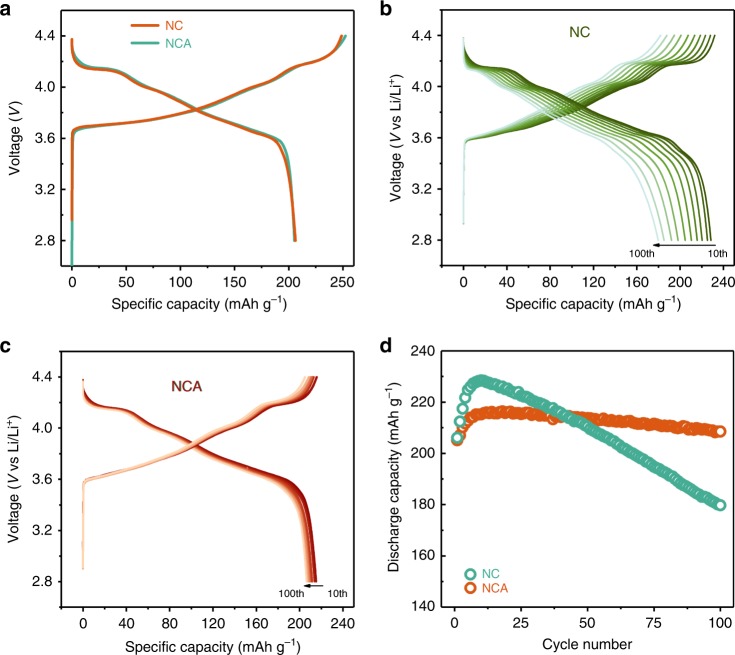
Electrochemical cycling performance of NC and NCA measured in half-cell. **a** The first charge–discharge cycle at the voltage between 2.8 and 4.4 V. The red and green lines represent the plots of NC and NCA, respectively. The charge–discharge profiles of **b** NC, **c** NCA, from 10th to 100th cycles. **d** Discharge capacity evolution as a function of cycle numbers. Each red and green ring represents the capacity of NCA and NC collected from 1st to 100th cycle

The electrochemical performance of NCA and NC are evaluated in a half-cell using the identical cycling parameters: cycling at 2.8–4.4 V, at the temperature of 25 °C, and at the rate of 0.2 C. For NC, the first cycle data in Fig. 2a demonstrates a low Coulombic efficiency of 82.9%, a typical value reported for NMC materials and is often ascribed to the loss of Li inventory^39–41^. The NCA shows a similar first cycle Coulombic efficiency of 81.3%, indicating the Al doping plays a negligible role in ameliorating the initial high inefficiencies. The beneficial roles of Al doping yet become evident as the cycling progresses. The NC group displays fast performance decay as typified by the decrease of 100 mV for voltage and 48.7 mAh^−1^ for the capacity from 10th to 100th cycles, as shown in Fig. 2b. The severe performance decline is observed to be effectively mitigated by Al doping, as evidenced by the significantly narrowed voltage drop and effectively retained capacity in the NCA group, with a value of 5 mV and 95% after 100 cycles (Fig. 2c). The performance evolution of the two groups is further demonstrated in the discharge capacity plot (Fig. 2d). Initially, the NCA exhibits a lower capacity during the first few cycles, however, a performance breakpoint appears—NC group starts to undergo abrupt capacity decays after 10 cycles, in contrast, the NCA renders a minor decline in capacity over the subsequent cycles, and the performance of NCA become superior to NC starting from 43 cycles. The electrochemical performance illustrates that the Al substitution improves the electrochemical cycability, in terms of both capacity and voltage.

### Spatial distribution of Al in NCA

**Fig. 3:**
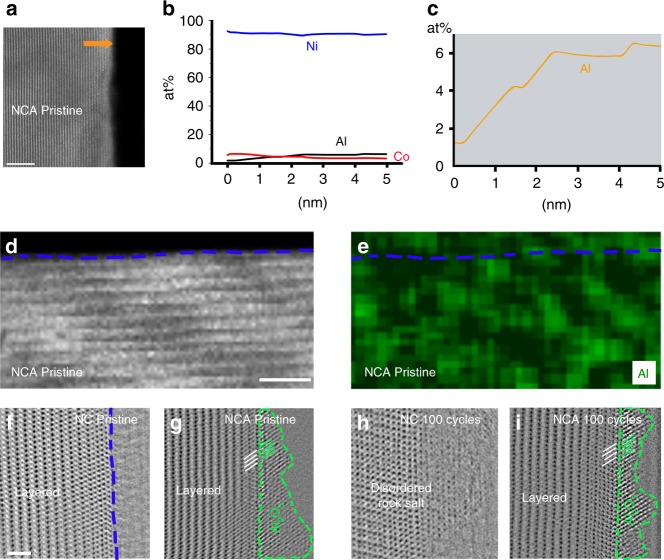
Spatial distribution of Al in NCA. **a** STEM-HAADF image of pristine NCA. **b** Atomic compositional profile of Ni, Co, and Al along the arrow in **a**. **c** The enlarged view of Al compositional profile, indicating enrichment of Al on the surfaces. HAADF-STEM image **d** and corresponding EDS mapping **e** of pristine NCA sample. The blue dashed lines represent the surface boundary of NCA primary particle, and the green color depicts the Al elements distribution. **f**, **h** The ABF images of pristine and 100 cycled NC surfaces, respectively. The blue dashed lines depict the boundary of NC surfaces. **g**, **i** The ABF images of pristine and 100 cycled NCA surfaces, respectively. The green dashed lines outline the Al_2_O_3_ phase. The white and green lines represent the (01$\bar 2$) planes of parent phase and ($\bar 1$20) planes of Al_2_O_3_ phase, respectively. Scale bar, 5 nm (**a**, **d**), 1 nm (**f**)

To provide mechanistic understanding of Al doping in improving the electrochemical performance, a thorough investigation of Al distribution and associated structure is essential. The EDS mapping reveals that the Al is partitioned into two typical parts: as bulk lattice solid solution and as Al_2_O_3_ particles. The scattered Al signals inside the bulk indicate the Al atoms are well incorporated into the NC parent phase (Fig. 3e), meanwhile, the cumulative signals obtained along the line profile drawn from surface towards bulk demonstrate a Al enrichment on surface, which is in line with reported phenomena of Al surface segregation in the layered structure^33,42^ and lead to the formation of core–shell like structure. That is, the core part has an Al concentration of <2 at% and the shell part possesses a Al concentration of ~6 at% (Fig. 3a–c).

Meanwhile, the comparison between STEM and the corresponding Al mapping images indicates that, in some local surface regions, the Al elements extend beyond parent NCA phase (the blue dashed lines in Fig. 3d and e), with a thickness of ~1–2 nm above surfaces. The annular bright field (ABF) imaging is used to identify the structure of the Al compound dressed on the primary particle surface, because the signals collected from the characteristic angles are sensitive to light elements. The captured images indicate that a second crystalline phase with distinct contrast indeed grows on top of NCA surfaces, with a thickness ranging from 1 nm to a few nanometers (Fig. 3g). This is further demonstrated by the comparison between HAADF and ABF images (Supplementary Fig. 3), the scenario that the second phase is visible in ABF while being absent in the HAADF indicates the newly developed phase is originated from light elements—Al, most likely to be the aluminum oxides. Moreover, the boundary of the second phase from imaging agrees well with the contour of Al and O from EDS mapping, confirming the Al compound to be Al_2_O_3_. The presence of Al_2_O_3_ compound is further confirmed by the comparison between pristine NC and NCA surfaces: the ABF image of pristine NC surface always presents a sharp flat boundary (Fig. 3f) that is coincide with the NC surface in HAADF images (Supplementary Fig. 4), however, a second contrast, with the peak and valley facets and located beyond the NCA surfaces, appears in the ABF images of NCA group (outlined by the green dashed lines in Fig. 3g), suggesting second phase composed of light elements –Al– are grown on the NCA surfaces. The semi-coherent interface between the Al compound and parent NCA phase reveals an epitaxial growth manner between NCA and Al_2_O_3_ with an orientation relationship of NCA[100]//Al_2_O_3_[841] and NCA (01$\bar 2$)//Al_2_O_3_ ($\bar 1$20) (Supplementary Fig. 5).

Al_2_O_3_ has been widely recognized to alleviate interface degradation and effectively improve the battery performance while being coated on NMC surfaces. Apparently the Al_2_O_3_ particles developed on NCA exhibit a similar effect as to the surface coating. Critically, the protection layers barely diminish on cycling, where the Al_2_O_3_ nano-islands present an invariant thickness of 1–2 nm after 100 cycles (Fig. 3i), indicating the Al_2_O_3_ particles are inert to the electrochemical and chemical reactions and may offer sustained protection through the prolonged cycling. The beneficial effects of Al_2_O_3_ layers are also partially revealed by the structure evolution in the ABF images, in contrast to forming thick rock salt (Fm$\bar 3$m) structure in the NC group (Fig. 3h), the NCA surface that is covered by the Al_2_O_3_ particles displays a configurations of minor cation mixing in Fig. 3i. Despite the well-recognized benefits of Al_2_O_3_-coating layers, its influences on the electrochemical performance are insubstantial due to the limited coverage of ~1% of overall surface.

### Enhanced interfacial stability of NC by Al lattice doping

**Fig. 4:**
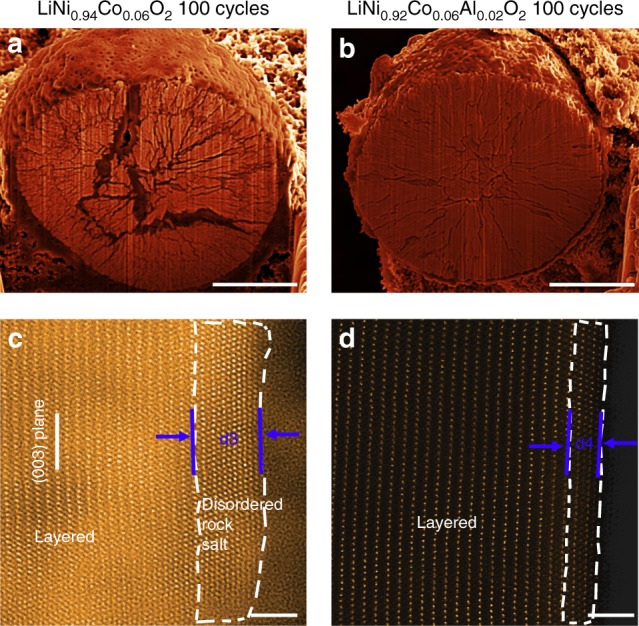
Secondary and primary particle morphological and structural evolution upon cycling. **a**, **b** SEM images of NC and NCA from cross-sectional view, showing the secondary particle pulverization after 100 cycles. **c**, **d** Phase transition on NC and NCA surfaces. The white dashed lines outline the contour of surface reconstruction layers. The d3 and d4 represent the thickness of disordered rock salt (Fm$\bar 3$m) layers and cation mixing layers in NC and NCA group, respectively. Scale bar, 4 μm (**a**, **b**), 2 nm (**c**, **d**)

The Al doping is characterized to significantly improve the electrode electrolyte interface stability. The cycling induced structure evolution of both NC and NCA materials are monitored at complementary scale in order to establish structure–performance correlation. The structure evolution of the secondary particle on cycling is examined by cross-sectional SEM. For the NC sample, the originally coherent, closely packed spheres suffer from severe disintegration during the course of electrochemical cycling, as shown in Fig. 4a, where large intergranular cracks nucleate and extend from the surface towards center, with the concomitant formation of significant amount of inter-granular cracks. In contrast, the NCA spheres are featured with only finer cracks and display no disintegration (Fig. 4b), indicating the Al-doping leads to the alleviation of particles pulverization. Therefore, the comparisons at macroscopic scale indicate one of the contributions from Al dopant is the reduced amount of inter-granular cracks.

Concurrently, the Al-induced structural modification is investigated at atomic scale by HAADF-STEM. In contrast to the similar surface configuration between the pristine primary particles of NC and NCA, a pronounced difference appears upon electrochemical cycling. The cycled NC primary particles show a core–shell structure, featuring a disordered rock salt phase of ~3 nm on the shell, as marked by d3 in Fig. 4c. By contrast, the NCA surface barely undergoes dynamic evolution upon cycling, as the very surface region presents a cation mixing layer with a thickness comparable to pristine NCA (Fig. 4d).

### Reduced transition metal dissolution in NCA

**Fig. 5:**
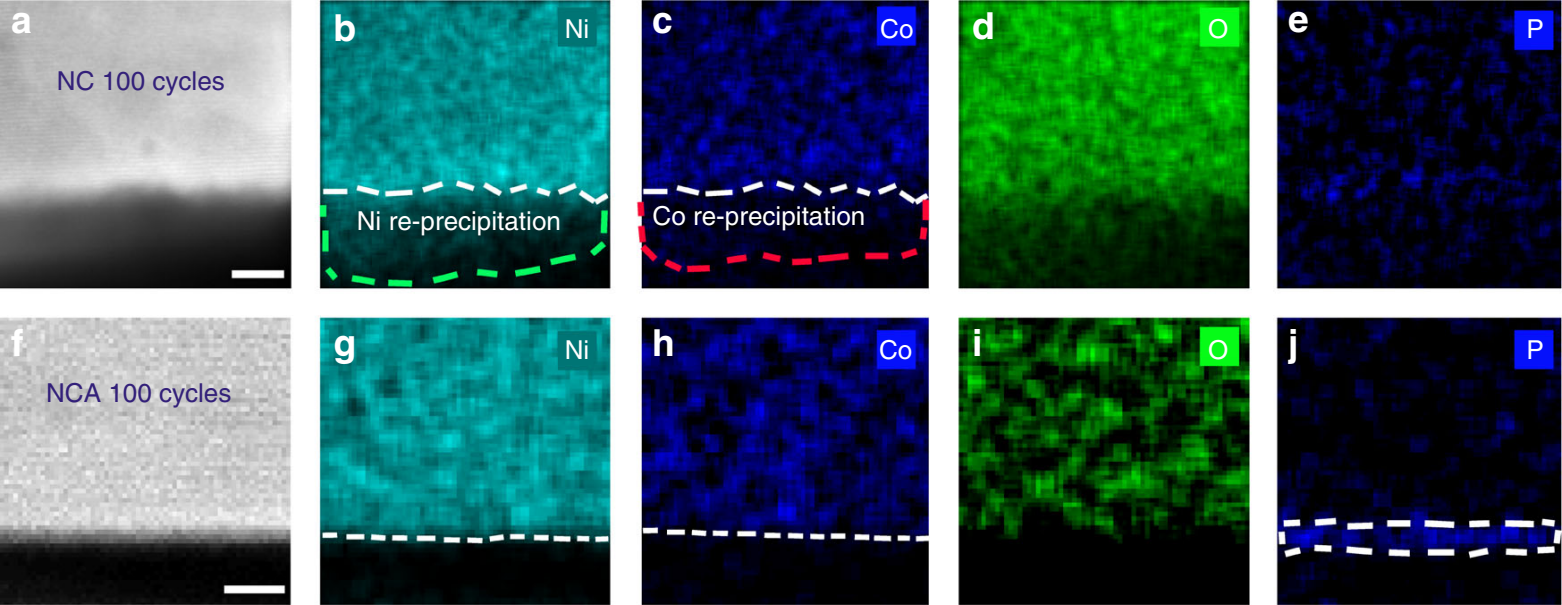
TM re-precipitation on NC surface and formation of P containing CEI layers in NCA samples. **a** HAADF-STEM image of NC sample after 100 cycles. **b**–**e** EDS mapping of Ni, Co, O, and P, respectively, from the region in **a**. The white lines outline the boundary of parent NC phase, and the green and red dashed lines outline the contour of amorphous Ni, and Co elements, respectively. **f** HAADF-STEM image of NCA surface after 100 cycles. **g**–**j** EDS mapping of Ni, Co, O, and P, respectively, from the region in **f**. The white lines in **g**, **h**, and **j** outline the boundary of Ni, Co, and P containing CEI layers, respectively. Scale bar, 3 nm (**a**), 4 nm (**f**)

Another beneficial effect of Al doping is the alleviated loss of active materials at the CEIs. The EDS mapping were performed at atomic scale to obtain comprehensive chemistry evolution during the course of electrochemical cycling. For both pristine NC and NCA samples, the native elements, such as Ni, Co, O, are uniformly distributed all over the parent phase (Supplementary Fig. 6). In addition, the layered structure always presents a characteristically sharp interface viewing along the [010] zone axis, indicating the primary particle surfaces are well-truncated. However, after 100 cycles, some blur contrast appears beyond the surface regions of NC group in the HAADF image (dash lines in Fig. 5a). Looking into the atoms arrangement reveals that blur phases are amorphous. The composition of the protruding phases is further confirmed by EDS mapping. The contour of Ni, Co, and O matches well with the boundary of amorphous phase in the HAADF image (Fig. 5b–d), moreover, the Ni intensity is significantly higher relative to Co, suggesting the re-precipitated compound is dominated by Ni accompanied with a small amount of Co (Supplementary Fig. 7). The TMs re-precipitation is much mitigated in NCA. The HAADF image demonstrates that the surface of NCA is well-reserved after 100 cycles, without presenting amorphous layers (white dashed lines in Fig. 5f). The EDS mapping results demonstrate that the Ni, Co, and O distribution is confined in the parent NCA regions (Fig. 5g–i), indicating the TMs dissolution and re-precipitation on NCA become negligible.

### Al doping-induced formation of stable CEI layers

For the major part of NCA surface without Al_2_O_3_ coverage, the protection layers are originated from CEI layers formation upon cycling. The EDS mapping reveals that a layer enriched with phosphate and with a thickness of ~3–4 nm is developed on NCA surface, as outlined by the white dashed lines in Fig. 5j. For the NC surfaces, even the apparent scattered P signals display in the EDS mapping (Fig. 5e), the spectrum from surface integration shows a negligible amount of phosphate, indicating the absence of P deposition at electrode electrolyte interface (Supplementary Fig. 8). The presence of P-enriched CEI layers is proposed to protect the cathode surface from corrosion and significantly improve the cycling behavior of NMC-layered materials^43^. The initial formation of CEI layers consumes active materials, leading to the irreversible capacity decay during the initial cycles as delineated by the electrochemical measurements (Fig. 2). The NCA shows a lower capacity relative to NC for the first 43 cycles. However, as the thin continuous CEI layers form, the adverse effects turn into positive and start to protect the interface, which make the NCA outperforms NC group with further cycling.

### Consequence of Al doping in NCA on anode

**Fig. 6:**
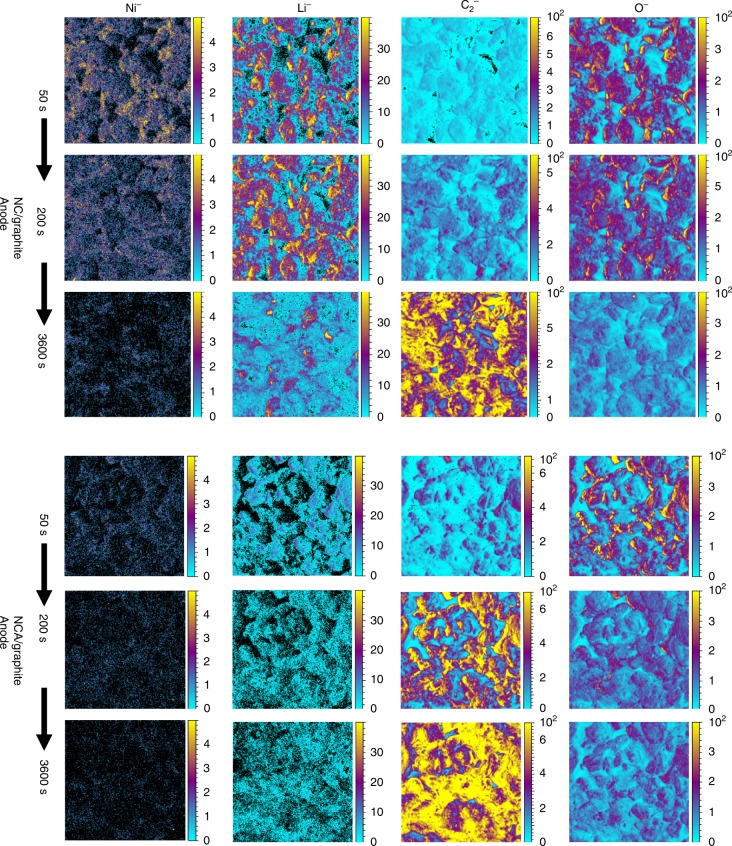
Anode SEI properties of NCA/graphite and NC/graphite cells. The scale bar labels the concentration of each segment using different colors. Each of the 1st, 2nd, and 3rd horizontal row represents the surface compositional mapping after 50, 200, and 3600 s sputtering, respectively. The 1st–4th vertical column represents the results of Ni^−^, Li^−^, C_2_^−^, O species

The solid electrolyte interface (SEI) properties on the anode often vary in response to the different surface reactivity on the cathode. A robust cathode surface prevents both the dissolution of transition-metals and the loss of active lithiums upon cycling, with concomitant better preservation of anode properties^44,45^. Therefore, the characteristics of SEI on the graphite anode can serve as an indicator for evaluating the robustness of cathode–electrolyte interfaces^10,46^. Because Li, C_2_, O, and Ni typically contribute to the formation of graphite SEI layers, the concentration and distribution of these species on the graphite surface were measured by time-of-flight secondary ion mass spectrometry (TOF-SIMS). Figure 6 shows the concentration evolution of each secondary-ion fragment as a function of sputtering time. For the NC sample, the TM dissolution is found to be more severe with respect to NCA, as evidenced by the higher concentrated and thicker Ni-layer deposition on the graphite counterpart (the first column in Fig. 6). The TMs, originated from cathode dissolution and cross talking, can catalyze the SEI formation and result in continuous electrolyte consumption, impedance build-up, and “dead” lithium accumulation^47,48^. As expected, the formation of “dead” lithium (represented by Li^−^ fragment in Fig. 6) is much retarded on the graphite surface when NCA was used as cathode in which a significantly thinner dead Li was identified for the NCA-graphite group (the second column in Fig. 6). Similarly, the depth profiling of C_2_^−^ signals points to the same conclusion: the thickness of SEI layers in NCA-graphite group is much thinner than that in the NC-graphite group. This conclusion is further supported by the O^−^ depth profiling by which O^−^ signal only comes from the decomposition of electrolyte (ethylene carbonate and vinylene carbonate (VC) in particular). After 200 s of sputtering, O^−^ signal remains strong and covers the majority of graphite surface in the NC group. In sharp contrast, O^−^ signals almost vanish for the NCA–graphite pair, which again indicates a much thinner SEI layer after Al doping.

## Discussion

Often, the detrimental effects associated with interfacial process originate from the penetration of electrolyte and proceed by the subsequent solid–liquid interfacial reactions. The processes start with the phase transitions on the primary particles surfaces in which the layered structure gradually transforms into the disordered rock salt phase, termed as the surface reconstruction, accompanied with the formation of reduced TM ions, e.g., Ni^2+^ on the primary particle surfaces. Without a barrier at the solid–liquid interfaces, the progressive growth of surface reconstruction layers can continuously contribute to the particle pulverization via two possible mechanisms: (1) The inherent lattice mismatch between the rock salt phase and layered structure inevitably generate internal strain, which has been proposed to be one of origins of inter-granular cracks formation^12,49,50^; (2) The layered-to-disordered rock salt phase transition converts the Ni^3+^ ions into Ni^2+^, which makes the Ni ions tend to dissolve and leads to the loss of active materials from the cathode particle surface. In addition, the dissolution of TM ions can drive the re-precipitation of TMs on the cathode surfaces, and more critically, on the anode via chemical crossover, which promotes the consumption of Li ions and expedites the growth of SEI layers.

The most effective means to prevent the cathode from degradation is to build barriers that can effectively suppress the side reactions at the solid–liquid interfaces. This is typically realized by the surface coating on the solid side, and by modifying the electrolyte chemistry on the liquid side. Based on our experimental observations, trace amounts of Al in the NC parent phase leads to two consequences: (1) the formation of Al-enriched shell. (2) The formation of Al_2_O_3_ nano-islands on top of NCA surface. Departure from the conventional manipulation of the electrolyte, the formation of P containing layers on the NCA surfaces, while absence in NC groups suggests another possible practice to alter the interface passivation layers by tuning the electrode, that is, facilitating the formation of thin CEI layers by Al doping. Despite both the CEI layers and the dressed Al_2_O_3_ layers are beneficial in protecting the interface, the contribution of Al_2_O_3_ nano-islands is minor because of the low coverage of Al_2_O_3_ layers (~1%, Supplementary Fig. 9). By contrast, the Al-concentrated shell-induced CEI layers act as the major barriers for the interfacial reactions.

**Fig. 7:**
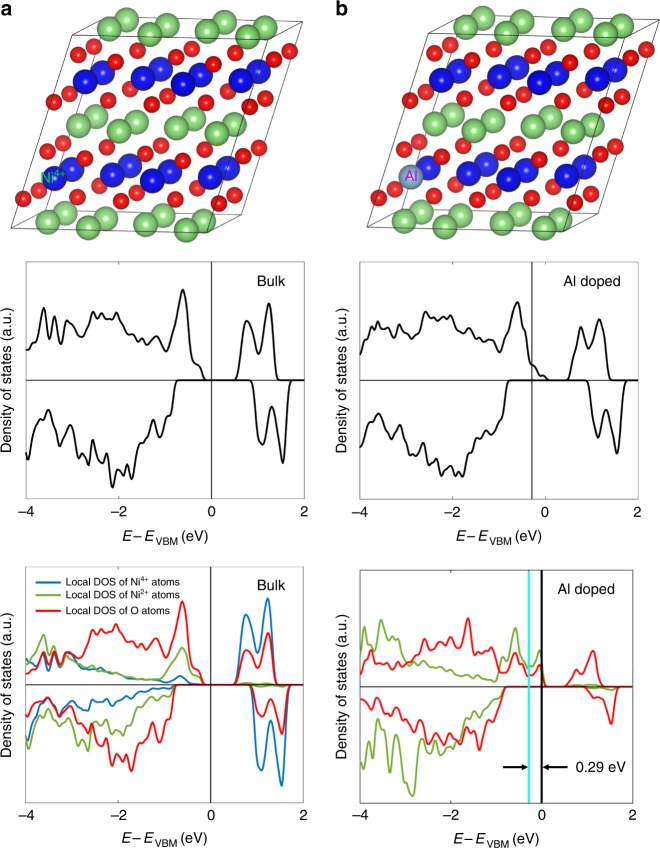
Al doping effects on the electronic structure of NC. *E*_VBM_ represents the maximum energy of valence bands of bulk NC. **a** Upper panel is the atomic model of bulk NC, middle and lower panels represent the total density of state and local density of states of each individual atom, respectively. The blue, green, sliver, and red balls represent the TM, Li, Al, and O atoms, respectively. **b** Upper panel represents the atomic model of Al-doped NC, in which the Al substitutes the Ni^4+^ atoms. Middle and lower panels represent corresponding total density of state and local density of states of each individual atom, respectively. The blue line represents the Fermi level after Al doping

Forming CEI layer through the oxidation of electrolyte is governed by the energy level of electrode relative to the electrolyte. The thermodynamic driving force for CEI layers formation would be readily provided when the Fermi level of electrode locates below the highest occupied molecular orbital (HOMO) of electrolyte. Often, the batteries are operated within the electrochemical window of conventional electrolytes, and thus the components of electrolyte are inert against thermodynamic oxidation. However, the electronic properties of TM oxides are usually varying in response to local environmental changes, for example, the redox potential differs as the evolution of TM oxidation state and the alteration of TM-anion covalency^51–53^. To investigate the effects of Al doping on the electrode electronic structure, we employ the DFT method to probe the density of state (DOS) of both undoped and doped electrodes. In a real cycled Ni-rich cathode, the charge for TMs varies with co-existence of Ni^2+^ and Ni^4+^ ions in many local environments. This is on one side caused by the inherent charge disproportionation from Ni^3+^, and on the other side originated form the oxidation state evolution associated with the lithiation and delithiation processes. Therefore, the structure with P2/c space group is adopted for the calculations because of its high accuracy in describing the charge properties^54^. Three unit cells were built: One is the pure Li transition metal compound with a mixture of Ni^2+^ and Ni^4+^ located on the side and center, the other two with the 6% Al substitution on the Ni^2+^ and Ni^4+^ site (based on the EDS measured results, Fig. 3a–c). The DOS plots suggest the Ni-rich compound possesses the semiconductor feature, with a bandgap width of 0.45 eV, and is consistent with the experimental measurements on LiNiO_2_^55^. Substituting Ni^2+^ ions with Al slightly modifies the bandgap width; however, no evident changes on *E*_f_ are observed (Supplementary Fig. 10). By contrast, an evident downshift of *E*_f_ with a value of ~0.29 eV displays for the substitution of Ni^4+^ sites (Fig. 7), which offers the possibility to make *E*_f_ approach HOMO and triggers the formation of thin and continuous surface passive films. This shows a qualitative consistency with our d*Q*/d*V* measurements in which the redox decrement of NCA is larger than that of NC upon cycling (Supplementary Fig. 11). Meanwhile, the calculations indicate that the substitution of Ni^4+^ are energetically more favorable than the Ni^2+^, because of the relatively lower overall energy (Supplementary Fig. 12), suggesting the Al substitution on Ni^4+^ sites contribute predominantly on the CEI layers formation. In addition, with the trace Al dopant, the Al segregation does not scale significantly with the bulk Al-dopant concentration. Our calculations indicate that the Al concentration on the surface merely changes from 7.4% to 7.7% when the bulk Al rises from 3% to 6% (Supplementary Figs. 13 and 14, Supplementary Table 2). Considering the substitution of Al can impede the Li diffusion, a least Al amount that triggers the CEI formation is expected to offer the best performance.

**Fig. 8:**
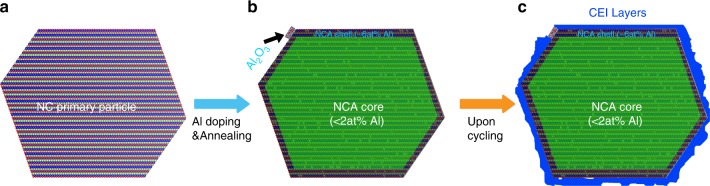
Schematic drawing of Al functioning mechanism in NCA. **a** Pristine primary particle of NC with layered structure. **b** The formation of core–shell structure and the development of Al_2_O_3_ nano-islands upon the high-temperature annealing during the materials synthesis stage. The green masked area indicates the core part with a Al concentration of <2 at% and the areas between the primary particle surface and green mask edge represent the shell part with a concentration of ~6 at%. The black arrow point to the Al_2_O_3_ nano-islands formed on top of NCA surface. **c** Formation of CEI layers upon the electrochemical cycling

In conclusion, through systematic comparison study of NC and NCA, we reveal that the dramatically improved cycling stability of NCA, as contrasted with that of NC, is originated from Al dopant enhanced cathode–electrolyte interface stability, due to the modifications of both structural stability and electronical energy of cathode. The improved interface behavior upon the prolonged cycling is characterized to be associated with several concurrent events: a much reduced TM dissolution and re-precipitation; a significantly thinner surface reconstruction layers; a less severe secondary particle pulverization; and a much reduced SEI thickness in the graphite anode. Two consequences originated from Al doping contribute to the improvement of interfacial stability: the development of epitaxial Al_2_O_3_ nano-islands (~1% of surface coverage) and NCA core–shell structure arising from the Al enrichment on the primary particle surface (Fig. 8b), which facilitates the formation of CEI layers upon cycling (Fig. 8c).The results extend the understanding of dopant effects from bulk to solid–liquid interface, and may be applicable in more general cathode systems.

## Methods

### Material preparation

Ni_0.94_Co_0.06_(OH)_2_ precursor with a particle size of ≈12 µm was prepared by transition-metal co-precipitation method. NiSO_4_·6H_2_O and CoSO_4_·7H_2_O with a molar ratio of 94:6 (Ni:Co) and a combined concentration of 2.0 M were firstly dissolved in distilled water. The mixed solution was then pumped into a 10 L batch tank reactor at a proper flowing rate. A second feed of solution containing a stoichiometric ratio of NH_4_OH and KOH was injected into the reactor to maintain the desired PH of ~11. The temperature was maintained at ~50 °C and stirring rate was set to be constant through the synthesis stage. The as-obtained powder was washed with distillated water several times, filtered by cellulose filter paper, and then dried overnight at 100 °C. Al(OH)_3_ and Ni_0.94_Co_0.06_(OH)_2_ were intermixed using a solution-based recipe. A proper amount of aluminum isopropoxide was dissolved in isopropanol, and then mixed with Ni_0.94_Co_0.06_(OH)_2_ precursor at 60 °C under vigorous stir till desiccation. The powder was further dried overnight at 100 °C before collection for future use.

For the preparation of LiNi_0.94_Co_0.06_O_2_, the obtained Ni_0.94_Co_0.06_(OH)_2_ precursor was mixed with lithium hydroxide monohydrate (Li:(Ni + Co) = 1:1.03 in mole), followed by calcinating at 500 °C for 3 h, and then at 640 °C for 12 h with flowing oxygen.

To prepare LiNi_0.92_Co_0.06_Al_0.02_O_2,_ the powder comprised of Al(OH)_3_ and Ni_0.94_Co_0.06_(OH)_2_ precursor was mixed with lithium hydroxide monohydrate (Li:(Ni+Co+Al) = 1:1.03 in mole). The mixture was firstly calcinated at 500 °C for 3 h, and then heated at 680 °C for 12 h.

### Electrode preparation and electrochemical characterization

Active material, Super P, and poly(vinylidene) fluoride (PVDF) with the weight ratio of 8:1:1 were dispersed in N-methyl-2-pyrolidone (NMP) and stirred to form uniform slurry. The obtained slurry was cast on an Al foil current collector with an active-material loading of ~5 mg cm^−2^. CR2032 coin cells were assembled inside an Ar-filled glove box (H_2_O < 0.1 ppm, O_2_ < 0.1 ppm). The electrolyte solution is comprised of 1.0 M LiPF_6_ in a mixture of ethylene carbonate/ethyl methyl carbonate (EC:EMC = 3:7 by weight) with 2 wt% VC. The half cells were cycled at a temperature of 25 °C, at a constant rate of *C*/5, and at between 4.4 and 2.8 V.

### TEM samples preparation and STEM characterization

The pristine NC and NCA powder and 100 cycled NC and NCA electrodes were harvested from the half cells, followed by washing with dimethyl carbonate (DMC). The as-prepared electrodes were then transferred to the Helios to do the lift out. A thin lamellar with a width of 3 μm was lifted out of the secondary particle of each sample using FIB, and then was attached on the Cu TEM grid using Pt. The lamellar was then thinned step by step using a voltage at 30 kV and polished at a lower voltage of 2 kV. The samples were then transferred to TEM for characterizations when they reach electron transparent thickness. The HAADF-STEM imaging and EDS mapping were performed on the aberration corrected JEOL JEM-ARM200CF, at the operation voltage 200 kV. The electrons from 90 to 370 mrad, and 10–23 mrad were collected for HAADF-STEM, and ABF-STEM imaging, respectively.

### Computational method

The electronic structures of LiNiO_2_ with and without Al doping were evaluated by means of density functional theory (DFT) calculations^56,57^. All the calculations were performed using the Vienna ab initio simulation package (VASP) code^58–61^. Projector augmented wave (PAW) pseudopotential was used to describe the electrons and a plane wave basis set with a kinetic energy cutoff of 500 eV was used to expand the wave functions. The generalized gradient approximation (GGA) in the Perdew, Burke, and Ernzernhof (PBE)^62^ formulation was employed to evaluate the electronic exchange and correlation. The Hubbard-*U* correction of *U*_eff_ = 6.4 eV was applied to the Ni *d* states^63–66^. The charge disproportionate ground state of LiNiO_2_ in P2/c symmetry was used in the current study, in which the valency of the Ni ions are half 2+ and half 4+ states^67^. The lattice parameters of LiNiO_2_ unit cell (shown in Supplementary Fig. 1) were determined to be *a* = 4.93328 Å, *b* = 5.79790 Å, *c* = 5.03578 Å, and *β* = 70.7698°. Al dopant was introduced into a 2 × 1 × 2 supercell by substituting either Ni^2+^ of Ni^4+^ ions. The Brillouin zone was sampled using Γ centered 4 × 6 × 4 *k*-point grids for structural relaxation, whereas the *k*-point density was tripled for the DOSs calculations. During the structural optimization, the atomic positions of all atoms were fully relaxed until the Hellmann–Feynman force acting on each atom was <0.01 eV Å^−1^.

## Supplementary information

Supplementary Information

## Data Availability

The data that support the findings of this study are kept at the Environmental Molecular Sciences Laboratory at Pacific Northwest National Laboratory and are available from the corresponding authors on request.
