# Supplementary material for: Lattice doping regulated interfacial reactions in cathode for enhanced cycling stability
Source: Nat Commun. 2019 Aug 1;10:3447. doi: 10.1038/s41467-019-11299-2 (PMC6673690; doi:10.1038/s41467-019-11299-2)
Supplement: Supplementary file 1 — Supplementary Information [file 41467_2019_11299_MOESM1_ESM.pdf]

Supplementary Information for

Lattice doping regulated interfacial reactions in cathode for enhanced cycling stability

Zou et al.

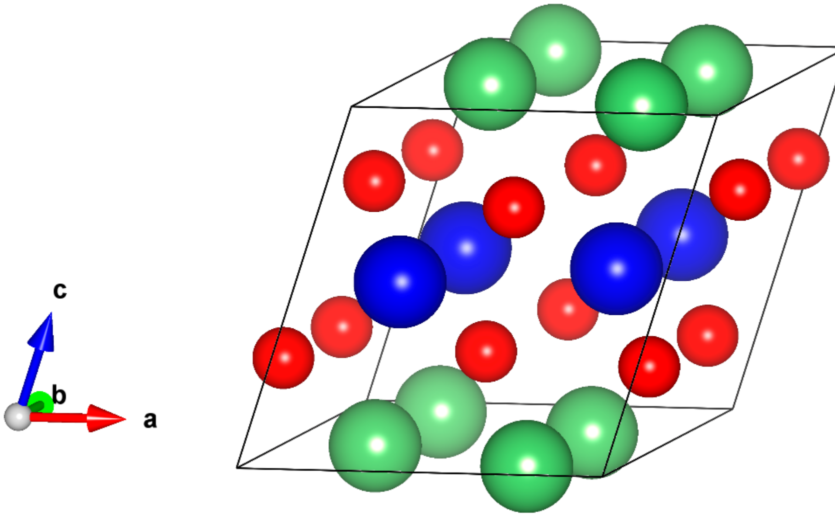

Supplementary Figure 1 | Unit cell of $LiNiO_2$ in P2/c symmetry. The blue, green, and red balls represent the transition metal, Li, and O atoms, respectively.

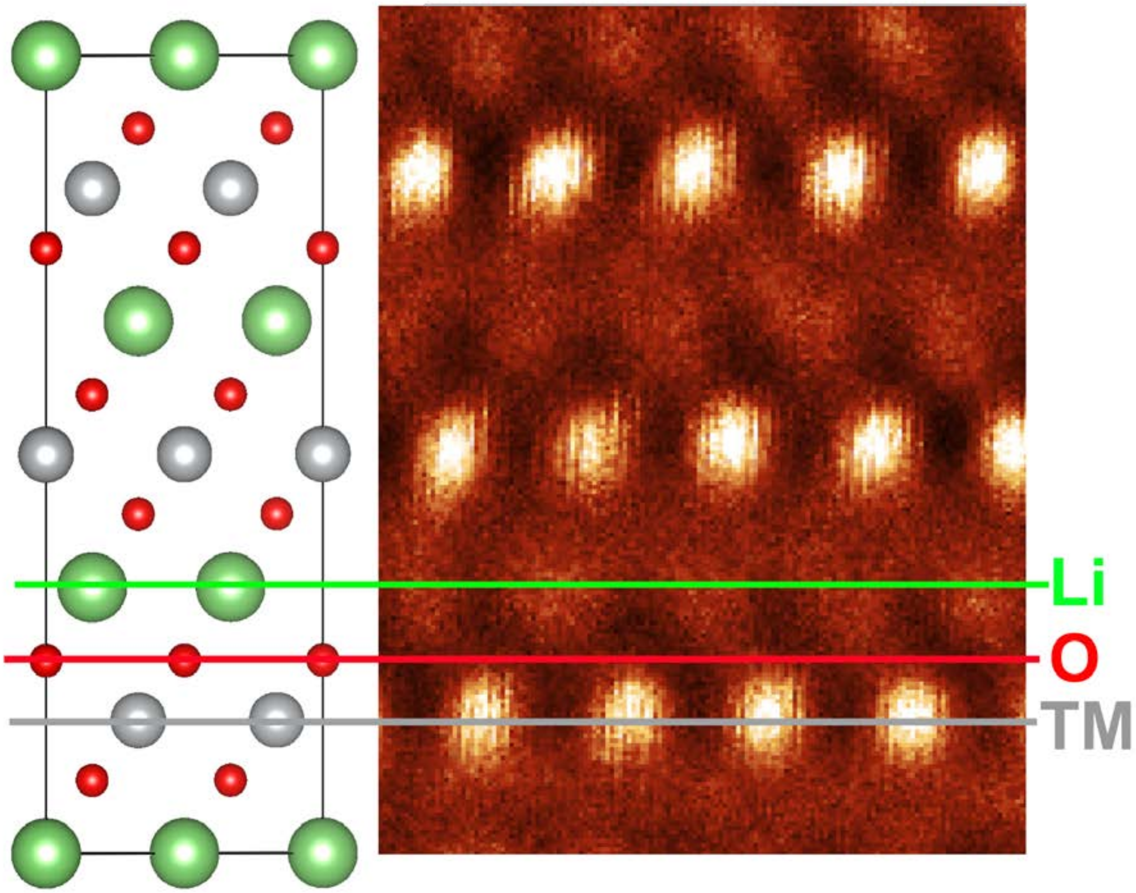

Supplementary Figure 2 | Location of Li, O, and TM in the layered structure. The green, red and grey lines mark the positions of Li, O, and transition meal (TM) row, respectively.

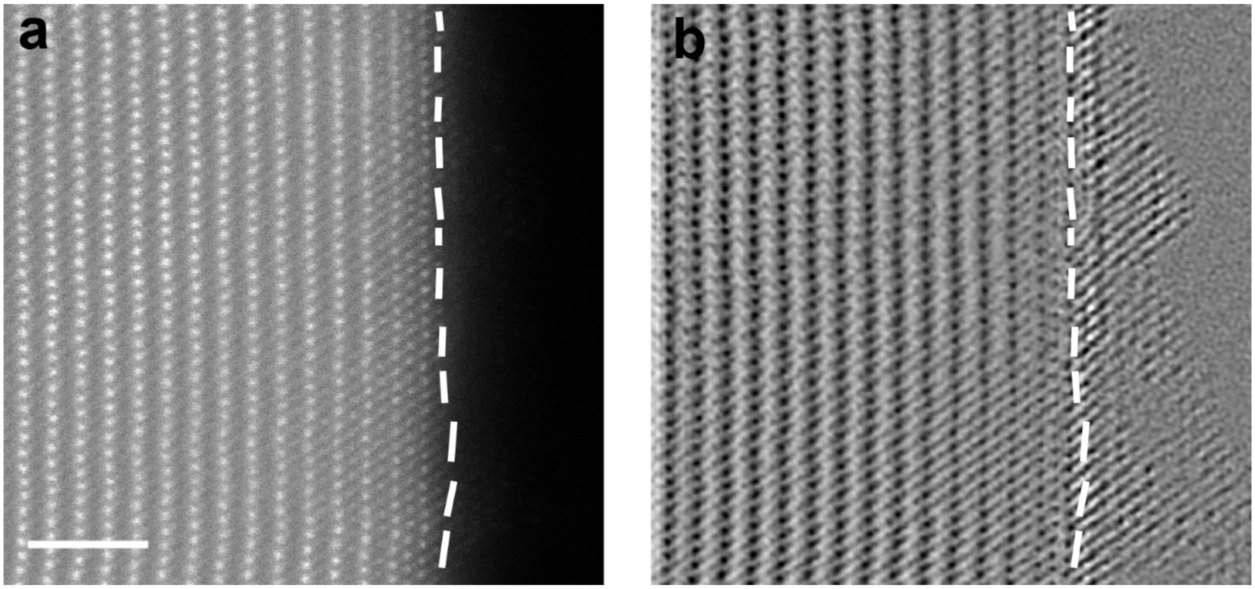

Supplementary Figure 3 | Comparison of HAADF and ABF images in pristine NCA materials. (a) HAADF and corresponding. **(b)** ABF image from surfaces of NCA. Scale bar 2 nm **a**

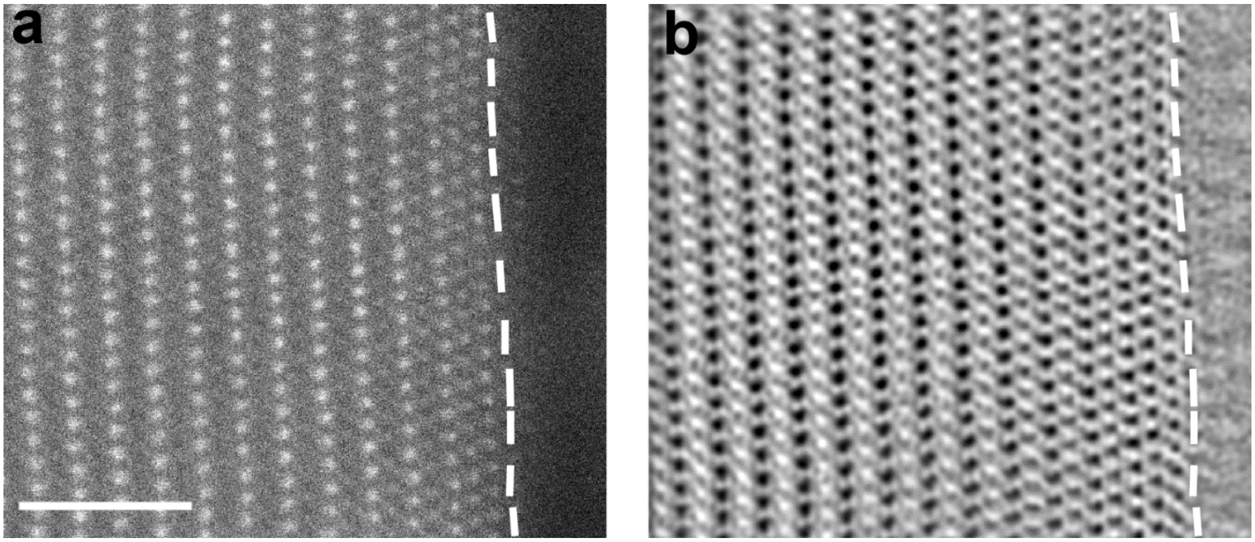

Supplementary Figure 4 | Comparison of HAADF and ABF images in pristine NC materials. (a) HAADF and corresponding. **(b)** ABF image from surfaces of NC. Scale bar 2 nm
a

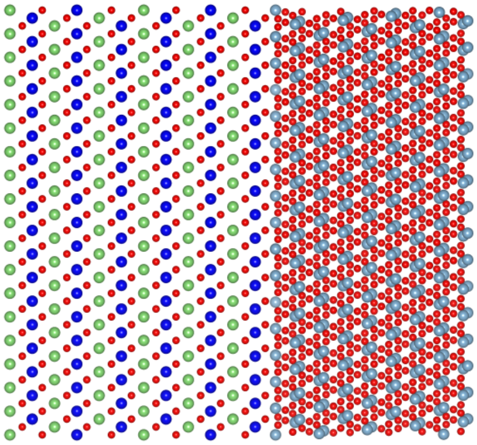

Supplementary Figure 5 | Structure model of NC and Al_2O_3 interface. The blue, green, red, and silver spheres represent the transition metal (TM), Li, O, and Al atoms, respectively.

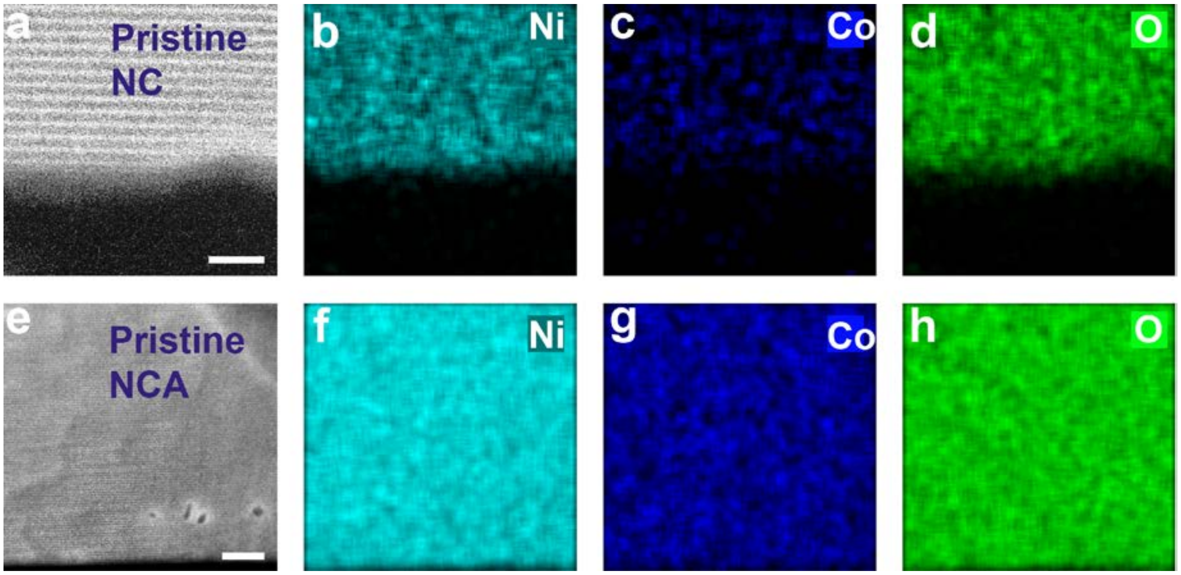

Supplementary Figure 6 | EDS mapping of the pristine NC and NCA materials. (a) HAADF-STEM image of pristine NC. **(b-d)** the corresponding mapping of Ni, Co, and O of **(a)**. **(e)** HAADF-STEM image of pristine NCA. **(f-h)** the corresponding mapping of Ni, Co, and O of **(e)**. Scale bar 2 nm **a**, 10 nm **e**

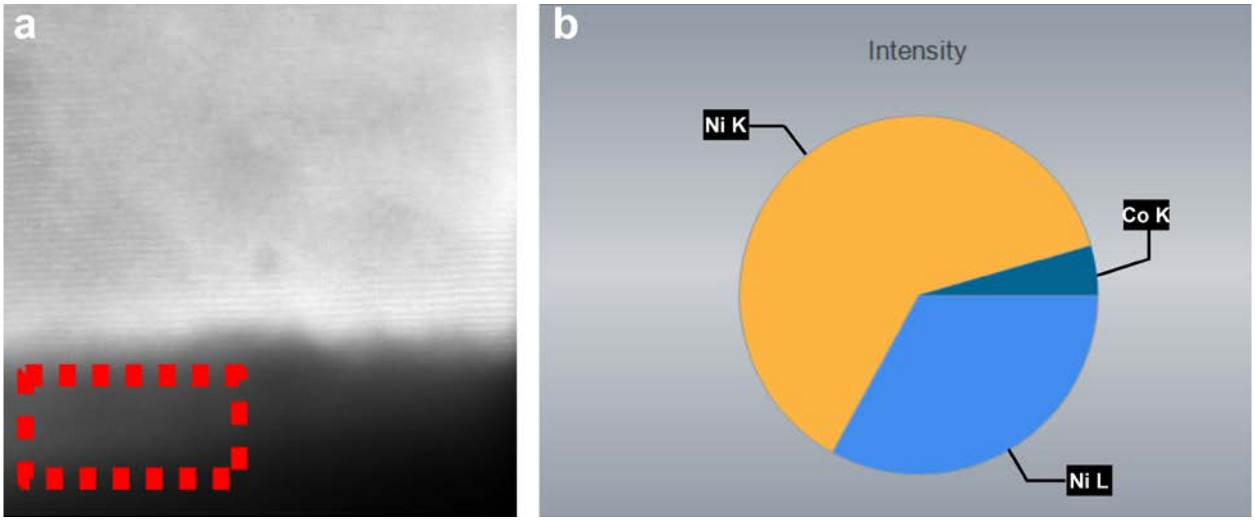

Supplementary Figure 7 | Composition of Ni and Co in the amorphous phase region. (a) HAADF-STEM image of 100 cycled NC surface. The dashed red rectangle outline the area for the EDS analysis. **(b)** The statistic percent of Ni K-edge, Ni L-edge, and Co K-edge from the rectangle area in **(a)**.

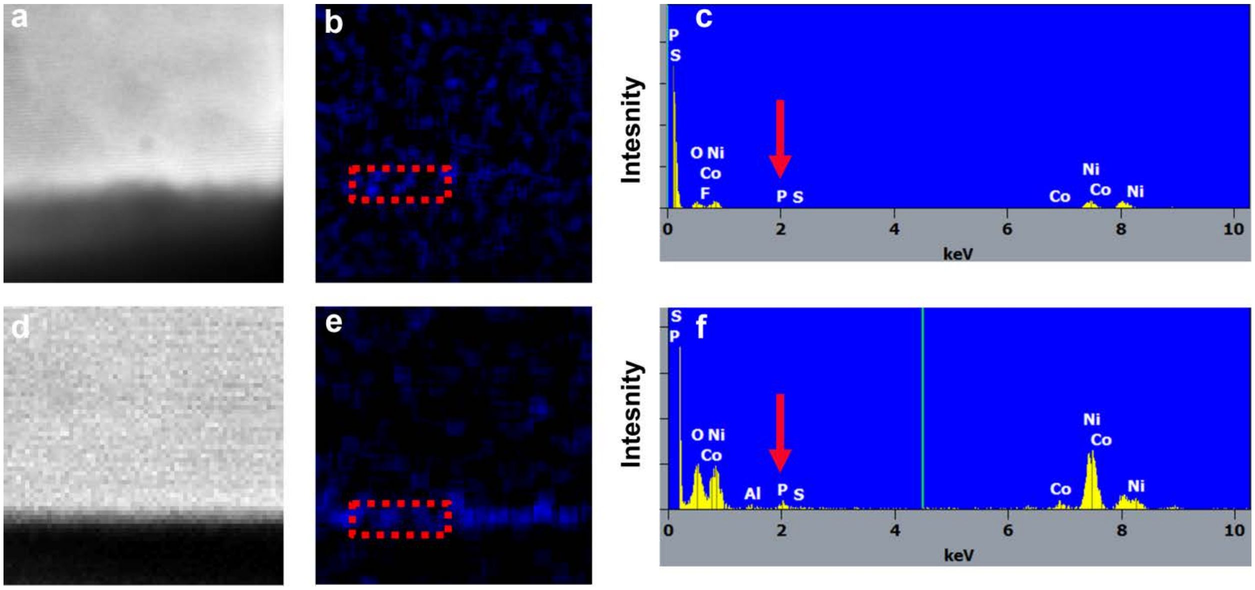

Supplementary Figure 8 | Phosphate spectrum of surface regions of NC and NCA. (a) HAADF-STEM image of 100 cycled NC. The dashed red rectangle outline the area for spectrum analysis. **(b)** Phosphate mapping of region in **(a)**. **(c)** Spectrum obtained from the region in **(a)**. **(d)** HAADF-STEM image of 100 cycled NCA. The dashed red rectangle outline the area for spectrum analysis. **(e)** Phosphate mapping of region in **(d)**. **(f)** Spectrum obtained from the region in **(d)**.

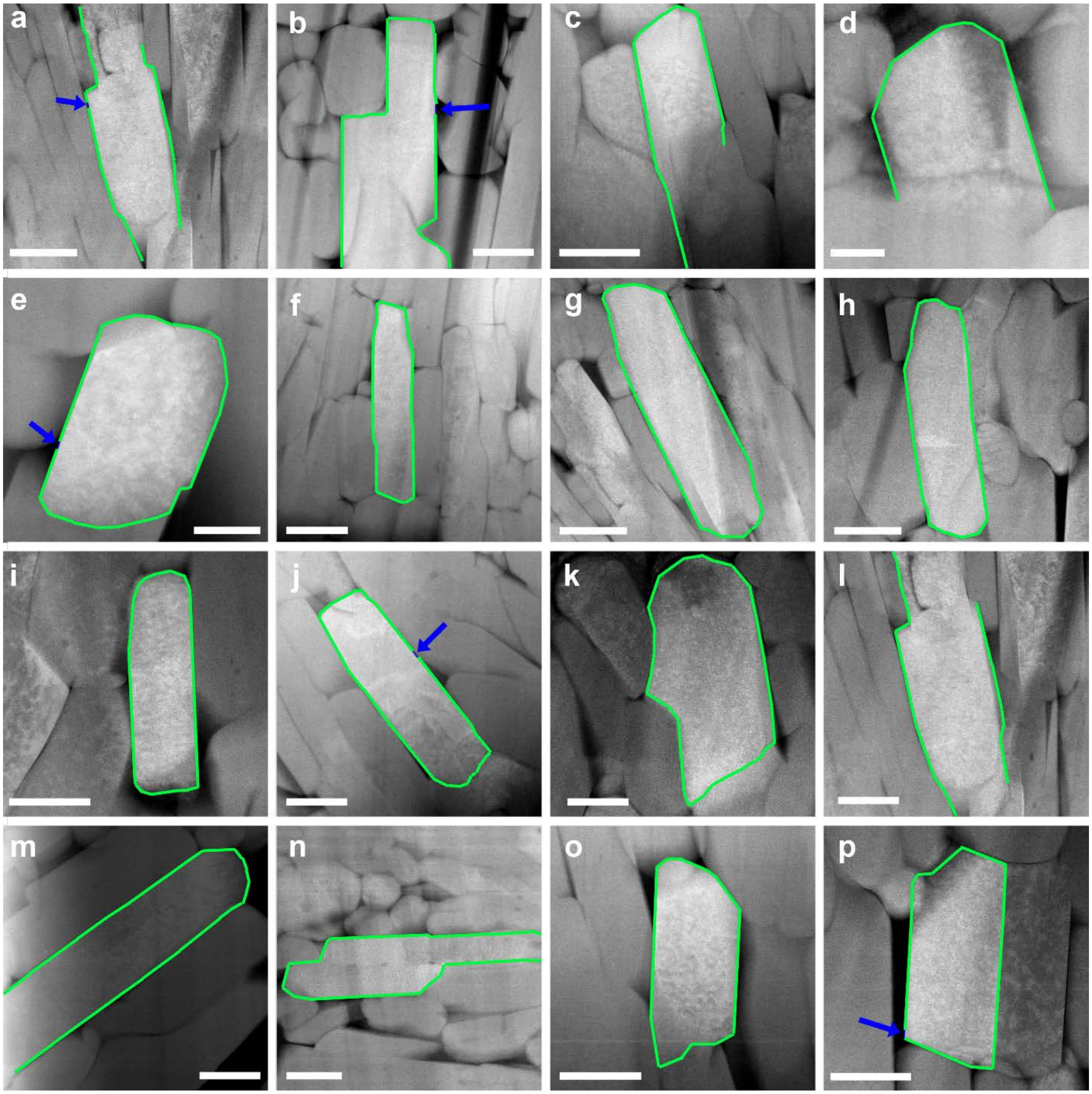

Supplementary Figure 9 | Al_2O_3 coverage on primary surfaces. (a-p) The analysis were performed on 16 primary particles. Each primary particle is rotated to [100] zone axis for high resolution imaging. The blue and green lines outline the surface regimes with and without Al_2O_3 coating layers, respectively. Scale bar 300 nm **f**. 200 nm **a, g, h, n**. 150 nm **(b-c), (j-l), m, p.** 100 nm **i, o**. 50 nm **(d-e)**

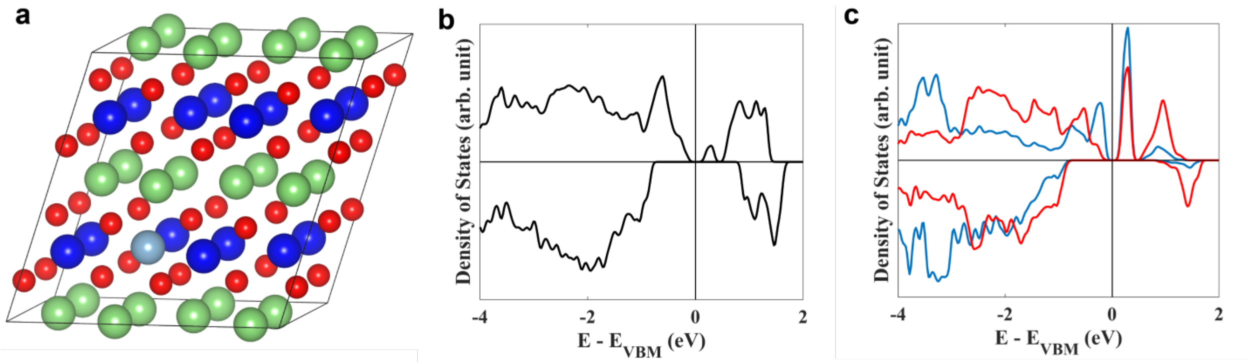

Supplementary Figure 10 | Electronic structure of Al substituted NC on Ni^{2+}. (a) Atomic model of Al doping on Ni^{2+} of bulk NC. **(b)** Total density of state of NCA. **(c)** Local density of states of each individual atom. Red and blue line represents the DOS of O and Ni^{2+} atoms.

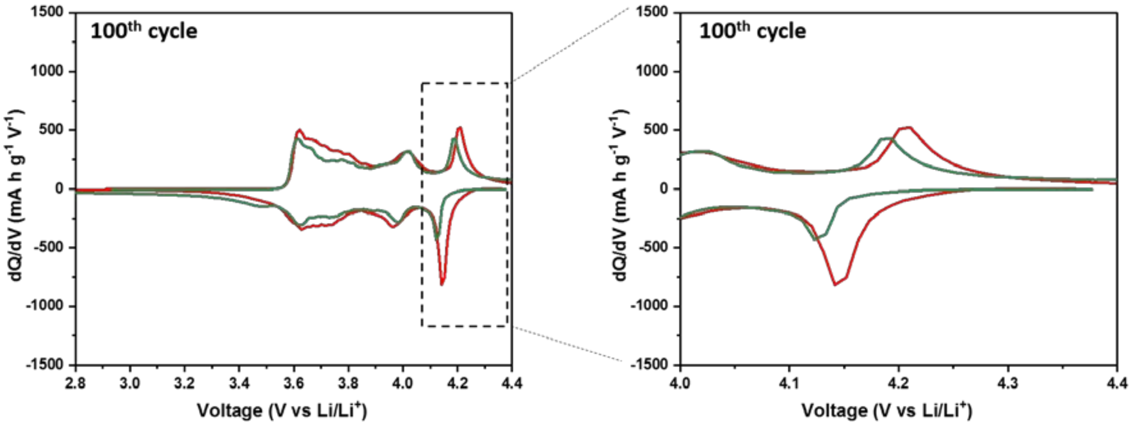

Supplementary Figure 11 | The dQ/dV plots of NC and NCA after 100 cycles. The right panel displays the zoom in view of the rectangle area in the left panel. The green and red lines represent the plot of NC and NCA respectively.

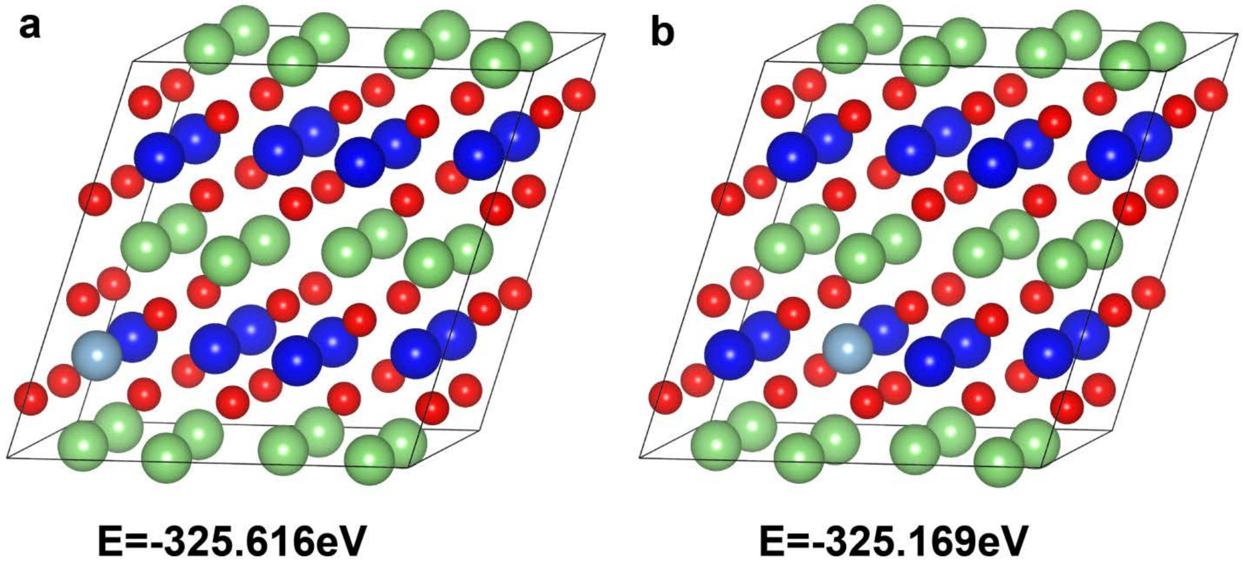

Supplementary Figure 12 | Energy of Al doped NCA. (a-b) structure and energy of Al substitution on Ni^{2+} and Ni^{4+}, respectively.

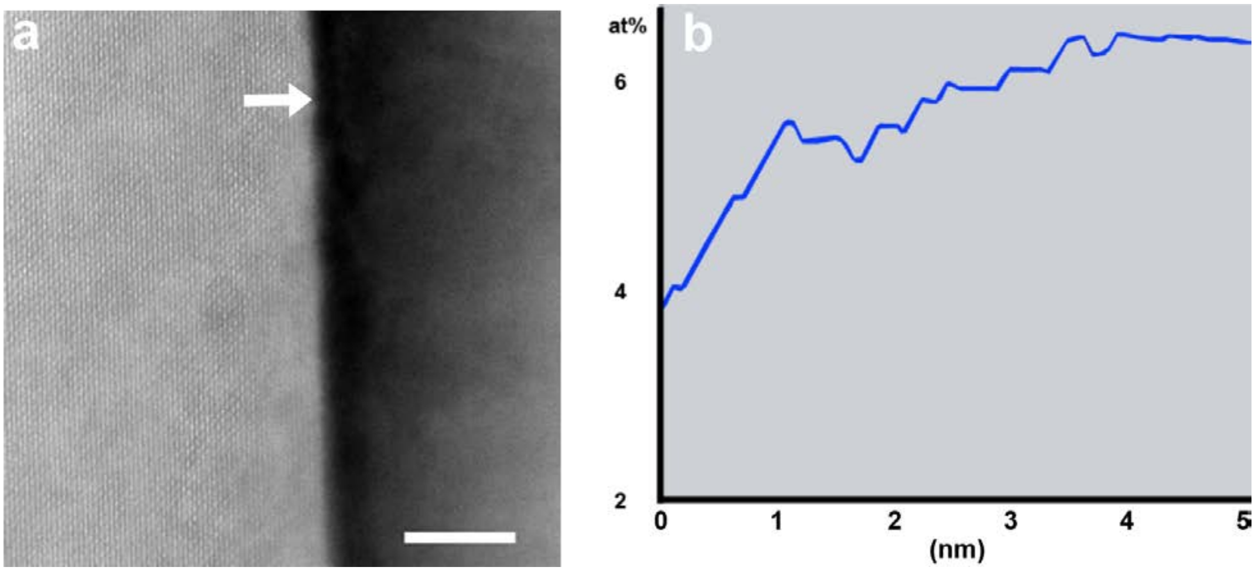

Supplementary Figure 13 | Al composition profile of NCA-4Al. (a) The area targeted for the compositional analysis. **(b)**The Al composition profile measured along the white arrow in **(a)**. Scale bar 10 nm **a**

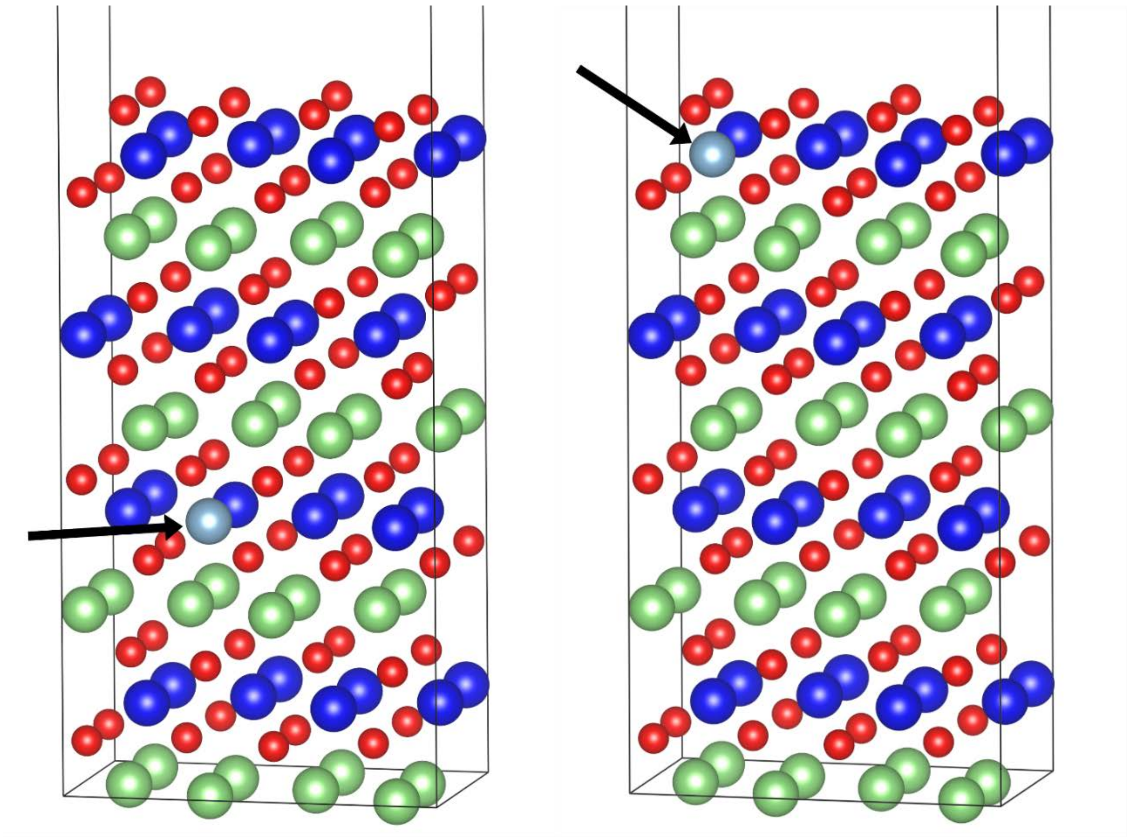

Supplementary Figure 14 | Calculation of Al surface segregation energy in NCA. The black arrows point to the Al atoms. **(a-b)** The atomic models with the Al atom locate in the bulk and surface, respectively.

Supplementary Table 1. Retrieved fitted results of pristine NC and NCA samples

$LiNi_{0.94}Co_{0.06}O_2$ (NC) (R-3m): a = b = 2.8712 Å, c = 14.1763 Å						
Atom	Position	x/a	y/b	z/c	Occupancy	U_{iso} (Å^2)
Li	3a	0.00000	0.00000	0.00000	0.900	0
Ni(2)	3a	0.00000	0.00000	0.00000	0.017	0
Ni(1)	3b	0.00000	0.00000	0.50000	0.923	0
Co	3b	0.00000	0.00000	0.50000	0.060	0
O	6c	0.00000	0.00000	0.24134	2.000	0.0106
R_p = 10.5, R_{wp} = 11.4, Chi2 = 4.8.						
$LiNi_{0.92}Co_{0.06}Al_{0.02}O_2$ (NCA) (R-3m): a = b = 2.8709 Å, c = 14.1858 Å						
Atom	Position	x/a	y/b	z/c	Occupancy	U_{iso} (Å^2)
Li	3a	0.00000	0.00000	0.00000	0.950	0
Ni(2)	3a	0.00000	0.00000	0.00000	0.037	0
Ni(1)	3b	0.00000	0.00000	0.50000	0.883	0
Co	3b	0.00000	0.00000	0.50000	0.060	0
Al	3b	0.00000	0.00000	0.50000	0.020	0
O	6c	0.00000	0.00000	0.24115	2.000	0.0104
R_p = 8.8, R_{wp} = 10.1, Chi2 = 7.2.						

Supplementary Table 2. DFT calculated results for NCA-3Al and NCA-6Al

	E_{bulk}	E_{surf}	E_{seg}	c_{bulk}	c_{surf}
1x1x4 (NCA-6Al)	−316.319	−316.336	−0.01681	0.0625	0.0767
2x1x4 (NCA-3Al)	−625.356	−625.427	−0.07063	0.03125	0.073848

Supplementary Note 1 | Statistic analysis of Al_2O_3 coverage

To obtain the statistical data of the Al_2O_3 coverage, we fibbed several different secondary particles using pristine NCA electrode. After that, we located different primary particles inside each secondary particle and zoomed in for high resolution imaging. The every surface regions is examined along the periphery of each primary particle, and the quantitative analysis is performed according to the projected edges. Our imaging results consistently show that only trace amount of Al_2O_3 is developed and covered on limited areas of NCA surfaces. The results of 16 primary particles are shown in Supplementary Figure 10, in which each of the primary particle is aligned to the [100] zone axis, and the every surface region is recorded at atomic resolution by simultaneously collecting the HAADF and ABF images at high magnification. Our imaging results show that most of surface regions are in the absence of the Al_2O_3 films (as outlined by the green lines in Supplementary Figure 10), and only a small portion of surfaces is covered by Al_2O_3 (indicated by the blue lines and arrows in Fig.10). The statistical data yields an average coverage of 1%, suggesting the minor coverage of Al_2O_3 coating on NCA surfaces.

Supplementary Note 2 | Redox decrement with Al doping.

Because Al concentrated shell is originated from the Al surface segregation, the regimes are only limited to the surface and subsurface regimes, and it only causes the significant Fermi level shift on these localized area, while the core part remains the concentration of <2% and does not present the significant shift. Note that the c-v curve displays the integrated signals averaged all over the electrode materials, including both the bulk and surface part. Given the considerably small volume of the Al segregated area, the bulk NCA is the dominant entity that contributes to the C-V curves. Therefore, the critical local redox decrement of surface regimes is likely not completely reflected on the ensemble-averaged plot. However, by checking the dQ/dV curves, the overall downshift trend of Fermi level is evident with Al doping, in which the average charge voltage decrement of NCA is 0.035V more than that of NC after 100 cycles, as shown in Supplementary Figure 12.

Supplementary Note 3 | Calculation of Al surface concentration as the Al bulk composition variation.

Two NCA models, one with the Al bulk concentration of 3.1% and the other with 6.2%, were firstly constructed to calculate the surface segregation energies. Here the surface segregation energy (E_{seg}) is defined by the energy difference between the model with Al locates in the bulk (Supplementary Figure 14a) and the one with Al resides on surface (Supplementary Figure 14b). Our calculated results yield a surface segregation energy of −0.017eV and −0.071eV for the NCA-3Al and NCA-6Al. Using the segregation energy as an input, we then employ the equation 1 to estimate the Al concentration on surface sites.

$$C_s=C_{bulk}*\exp(-E_{seg}/kT) \qquad (1)$$

Where the C_{bulk} and C_s represent the Al concentration on the bulk and surface, respectively. Here, the $T=680^oC$ is set for the calculations because the NCA synthesis is performed at such

temperature. This yields an Al surface concentration of 7.4% and 7.7% for the NCA-3Al and NCA-6Al (Supplementary Table 2), which is close to our measured results.
